# Genetic and Psychosocial Predictors of Aggression: Variable Selection and Model Building With Component-Wise Gradient Boosting

**DOI:** 10.3389/fnbeh.2018.00089

**Published:** 2018-05-07

**Authors:** Robert Suchting, Joshua L. Gowin, Charles E. Green, Consuelo Walss-Bass, Scott D. Lane

**Affiliations:** ^1^Department of Psychiatry and Behavioral Sciences, McGovern Medical School, University of Texas, Houston, TX, United States; ^2^Section on Human Psychopharmacology, National Institute on Alcohol Abuse and Alcoholism, Rockville, MD, United States; ^3^Center for Clinical Research & Evidence-Based Medicine, Department of Pediatrics, McGovern Medical School, University of Texas, Houston, TX, United States

**Keywords:** aggression, FKBP5, trauma, psychopathy, boosting, machine learning, data science

## Abstract

**Rationale**: Given datasets with a large or diverse set of predictors of aggression, machine learning (ML) provides efficient tools for identifying the most salient variables and building a parsimonious statistical model. ML techniques permit efficient exploration of data, have not been widely used in aggression research, and may have utility for those seeking prediction of aggressive behavior.

**Objectives**: The present study examined predictors of aggression and constructed an optimized model using ML techniques. Predictors were derived from a dataset that included demographic, psychometric and genetic predictors, specifically FK506 binding protein 5 (FKBP5) polymorphisms, which have been shown to alter response to threatening stimuli, but have not been tested as predictors of aggressive behavior in adults.

**Methods**: The data analysis approach utilized component-wise gradient boosting and model reduction via backward elimination to: (a) select variables from an initial set of 20 to build a model of trait aggression; and then (b) reduce that model to maximize parsimony and generalizability.

**Results**: From a dataset of *N* = 47 participants, component-wise gradient boosting selected 8 of 20 possible predictors to model Buss-Perry Aggression Questionnaire (BPAQ) total score, with *R*^2^ = 0.66. This model was simplified using backward elimination, retaining six predictors: smoking status, psychopathy (interpersonal manipulation and callous affect), childhood trauma (physical abuse and neglect), and the FKBP5_13 gene (rs1360780). The six-factor model approximated the initial eight-factor model at 99.4% of *R*^2^.

**Conclusions**: Using an inductive data science approach, the gradient boosting model identified predictors consistent with previous experimental work in aggression; specifically psychopathy and trauma exposure. Additionally, allelic variants in FKBP5 were identified for the first time, but the relatively small sample size limits generality of results and calls for replication. This approach provides utility for the prediction of aggression behavior, particularly in the context of large multivariate datasets.

## Introduction

Aggression is a complex multifaceted phenomenon (Anderson and Bushman, 2002; Raine, 2002; Mendes et al., 2009) that is influenced by many factors. Understanding and prediction of aggression must account for this complexity in order to extract a meaningful signal from amidst considerable noise. Key factors include: developmental history—notably childhood trauma (Caspi et al., 2002; Gowin et al., 2013; Milaniak and Widom, 2015) presence of psychopathology (Glenn and Raine, 2009; Alcorn et al., 2013; Anderson and Kiehl, 2014); externalizing personality traits (Gardner et al., 2015; Pasion et al., 2017); emotional and inhibitory dysregulation (Gao et al., 2015; Coccaro et al., 2016; Hsieh and Chen, 2017); biological factors, including genetic variation (Tuvblad and Baker, 2011; Bevilacqua et al., 2012; Takahashi et al., 2012; Dorfman et al., 2014); and contextual/situational factors such as substance use and provocation (Miczek et al., 2002; Cherek et al., 2006; Giancola et al., 2009; Skibsted et al., 2017).

Science has traditionally progressed via isolation of and emphasis on individual variables in the tradition of hypothesis testing and frequentist statistical inference, while fewer studies have utilized discovery-based, data science approaches in the study of aggressive behavior (but see Ang and Goh, 2013; Carré and Olmstead, 2015; Rosellini et al., 2016). As data science has become more established and widely utilized in scientific discovery and prediction (Hastie et al., 2009; Hofman et al., 2017; Wiens and Shenoy, 2018), novel inductive analytic techniques have enabled and advanced the analysis of complex, multivariate data. These approaches include mining of very large datasets, as well as application to smaller datasets where large amounts of information are obtained from each individual, but the dataset contains a relatively small number of subjects. In the present study, we utilized a data science approach to examine predictors of trait aggression, including interpersonal and demographic variables, history of trauma, psychopathology and genetic variations in the FK506 binding protein 5 (FKBP5) protein.

The FK506 binding protein 51 (FKBP5) is a glucocorticoid-related chaperone and immunophilin protein that plays a role in immune system function. Relevant to the present report, FKBP5 is implicated in emotional dysregulation. Specifically, certain FKBP5 variants appear to modulate clinically relevant aspects of mood and behavior in the context of childhood trauma and post-traumatic stress disorder (Klengel et al., 2013; Klengel and Binder, 2015; Zannas et al., 2016), as well as other stress-related pathologies via interaction with the glucocorticoid receptor (Bevilacqua and Goldman, 2011; Zannas et al., 2016). For example, FKBP5 gene × environment interactions play a role in depression (Gillespie et al., 2009; Appel et al., 2011; Tozzi et al., 2016), and—relevant to the present report—aggressive behavior in children (Bevilacqua et al., 2012; White et al., 2012; Bryushkova et al., 2016). Importantly, genetic variation for FKBP5 has not been tested as a predictor of aggressive behavior in adults. Thus, we examined three FKBP5 single nucleotide polymorphisms (SNPs) commonly implicated in stress-related emotional dysregulation.

As described above, variable selection for the present study was governed by factors with known associations to anger, inhibitory control, and aggressive behavior. However, our data science-informed analytic approach (described below) should be understood as quasi-exploratory rather than driven by traditional hypothesis testing. The primary goals were to: (1) determine which of the known predictors of aggression were most important; and (2) to examine the contribution of a hypothesized genetic variant toward trait aggression. Machine learning (ML) was used to explore these goals without overfitting the trait aggression outcome, measured here by the Buss-Perry Aggression Questionnaire (BPAQ; Buss and Perry, 1992).

## Materials and Methods

### Participants

Forty-eight participants were recruited from the greater Houston metropolitan area using local newspaper and radio advertisements, as part of a larger experimental study described in Gowin et al. (2013) and summarized below. This study was carried out in accordance with the recommendations of the Belmont Report and the University of Texas Health Science Center Committee for the Protection of Human Subjects (IRB), with written informed consent from all subjects. All subjects gave written informed consent obtained in person in accordance with the Declaration of Helsinki. The protocol was approved by the University of Texas Health Science Center Committee for the Protection of Human Subjects. For the present analyses, participants provided demographic information, psychometric data and saliva samples. *K*-nearest neighbors imputation was used to fill in a small amount (<2.5%) of missingness in the data on the child trauma questionnaire (CTQ) and Shipley II predictors.

### Design

The present study was derived from a larger, laboratory-based experimental study in which adult participants were given acute dose of 20 mg cortisol or placebo, and measures of salivary cortisol and state aggression (Point Subtraction Aggression Paradigm) were taken over a 5-h testing period (Gowin et al., 2013). To increase the likelihood of including participants with histories of trauma and heightened aggression, we advertised for individuals on parole or probation. We have used this strategy in several previous studies of childhood trauma and/or aggression (Gowin et al., 2010, 2013; Alcorn et al., 2013). However, we did not specify participant selection based on any DSM diagnostic and psychometrically-established clinical cut-offs for trauma exposure. In addition to the experimental procedures, measures of trait aggression, childhood trauma, and psychopathy were obtained at baseline from all participants. Additionally, at baseline a subset of 48 participants provided demographic information and saliva samples for genetic testing focused on FKBP5 Summarized below, the baseline measures collectively formed the dataset for the present analyses.

### Measures

#### Demographics

Following from established associations described in the introduction and based on baseline demographic variables collected in the Gowin et al. (2013) study age, education, ethnicity, sex and smoking status were included as demographic predictors in the present study.

#### Buss-Perry Aggression Questionnaire (BPAQ; Buss and Perry, 1992)

This measure of aggression features four subscales derived from factor analysis: physical aggression, verbal aggression, hostility and anger. It is a widely used psychometric measure of aggression, employed across a range of contexts and populations of interest. The dependent variable used in the present analyses was BPAQ total score, calculated by summing the standardized scores on the constituent subscales of the BPAQ. The BPAQ has strong psychometric properties (Buss and Perry, 1992; Harris, 1997), and use of the total score is established in previous studies of aggression (Moeller and Dougherty, 2001; Palmer and Thankordas, 2005; Gowin et al., 2013). The sum of the four factor scores results in a total aggression score. The BPAQ total score was used as the primary outcome.

#### Child Trauma Questionnaire (CTQ; Bernstein and Fink, 1998)

The CTQ is a 28 item self-report Likert-type scale of maltreatment during childhood. The instrument consists of five subscales: physical abuse, physical neglect, emotional abuse, sexual abuse and emotional neglect). The CTQ is a 28 item self-report Likert-type scale of maltreatment during childhood. The instrument consists of five subscales: physical abuse, physical neglect, emotional abuse, sexual abuse and emotional neglect). It is perhaps the most common psychometric instrument used in the measurement of childhood trauma exposure (Viola et al., 2016).

#### Impulsive/Premeditated Aggression Scale (IPAS; Stanford et al., 2003)

The impulsive/premeditated aggression scale (IPAS) is a 30 item self-report measure that classifies aggression into two sub-scales, premeditated and impulsive. It has measurement sensitivity related to history of violence, trauma and aggression-related personality characteristics (Stanford et al., 2008; Teten et al., 2008). Scores from the two subscales were used as independent predictors in the present analysis.

#### Self-Report Psychopathy Scale III (SRP-III; Neumann et al., 2012)

The self-report psychopathy scale III (SRP-III) is a Likert-type scale of psychopathy, measured on a scale from 1 to 5. The measure consists of four subscales: callous affect, erratic lifestyle (ELS), criminal tendencies and interpersonal manipulation. The instrument is sensitive in both normative samples and populations with externalizing psychopathology related to aggression (Alcorn et al., 2013). Scores from each subscale were used as independent predictors in the present analysis.

#### Shipley II Test of Cognitive Aptitude (Shipley et al., 2009)

The Shipley II is a measure of cognitive aptitude that correlates highly with general intelligence scales. The test construction used in the present study consisted of one 40-item verbal subscale (vocabulary) and one 20-item non-verbal subscale (block patterns). A composite score is derived from the two subscales and provides an index of overall cognitive ability. The composite score was used in the present data analyses.

#### FK506 Binding Protein 5 (FKBP5 Gene)

Genomic DNA was extracted from saliva Oragene DNA collection kits using the prepIT DNA extraction kit (DNA Genotek Inc, Ottawa, ON, Canada). Allelic discrimination for the FKBP5 SNP was performed using the Taqman 5’nuclease assay (Life Technologies, Grand Island, NY, USA). All samples were run in duplicate. Genotypes were determined using the ABI 7900HT SDS 2.2.2 software adapted in the ABI 7900HT Sequence Detection System. Based on previous work outlined in the introduction, the following SNPs were examined: FKBP5_13 (rs1360780); FKBP5_92 (rs9296158); and FKBP5_94 (rs9470080).

### Data Analytic Strategy

The present analysis utilized component-wise gradient boosting to develop an optimal model to predict aggression from the baseline set of 20 predictors (see Table 1). The optimal model was then simplified to maximize parsimony using a process called model reduction. Details of these techniques follow. All predictors were standardized by z-score before analysis to place them on a comparable metric and provide estimates of the relative influence of the predictor variables. The trait aggression outcome was left in its raw unstandardized metric to ease interpretability in raw units of the BPAQ score. This two-stage model building process has shown success in determining the best predictors of smoking lapse during a quit attempt (Suchting et al., 2017) as well as choosing the strongest inflammatory markers predicting depression in adolescents over time (Walss-Bass et al., 2018).

**Table 1:** Summary of all variables that served as candidate predictors (base-learners) in the initial component-wise gradient boosting (mboost) model.

Predictor variable	Frequency (%)
Sex	Male = 36 (75.00); Female = 12 (25.00)
Ethnicity	AA = 37 (77.08); Asian = 2 (4.17); Cauc = 3 (8.33); Hisp = 4 (10.42)
Smoking Status	No = 28 (58.33); Yes = 20 (41.67)
FKBP5_13	C/C = 22 (45.83); C/T = 18 (37.50); T/T = 8 (16.67)
FKBP5_92	C/C = 18 (37.50); C/T = 21 (43.75); T/T = 9 (22.92)
FKBP5_94	C/C = 16 (45.83); C/T = 21 (37.50); T/T = 11 (16.67)
	**Mean (SD) | Median (IQR)**
Age	31.69 (7.60) | 31 (13.00)
Education	13.84 (4.29) | 12 (2.00)
IPAS—Premeditated Aggression	20.96 (5.36) | 22 (6.50)
IPAS—Impulsive Aggression	27.66 (6.59) | 28 (9.00)
CTQ—Emotional Abuse	8.29 (3.48) | 7.5 (4.25)
CTQ—Physical Abuse	8.24 (3.16) | 8 (3.00)
CTQ—Sexual Abuse	6.22 (3.63) | 5 (0.00)
CTQ—Emotional Neglect	8.73 (3.64) | 6 (3.00)
CTQ—Physical Neglect	6.69 (1.94) | 5 (3.00)
SRP-III—Interpersonal Manipulation	38.62 (9.88) | 40 (14.50)
SRP-III—Callous Affect	41.98 (8.12) | 42 (10.50)
SRP-III—Erratic Lifestyle	42.87 (7.35) | 42 (10.00)
SRP-III—Criminal Tendencies	36.36 (10.86) | 36 (14.00)
Shipley II	200.27 (26.36) | 199 (40.00)
BPAQ—Total Score	64.04 (19.78) | 59 (22.50)

*Frequencies (%) and mean (SD) are provided for each predictor*.

#### Component-Wise Gradient Boosting

Component-wise gradient boosting is a ML technique for statistical model estimation that iteratively builds a strong prediction model from an ensemble of weak prediction models via gradient descent (Bühlmann and Hothorn, 2007). The technique seeks to model the relationship between some outcome (here, aggression) and a set of predictors using an algorithm that optimizes a loss function (e.g., for generalized linear models, the negative log-likelihood function). This algorithm is implemented in the mboost package in R (Hofner et al., 2014; Hothorn et al., 2016). In brief, the algorithm works as follows: (1) initialize an estimate of a function to fit the outcome with offset values; (2) specify a set of “base learners” (simple regression estimators); (3) compute the negative gradient of the loss function, fit each of the base learners separately to the negative gradient vector, select the best-fitting base-learner, and update the current function estimate with a shrinkage penalty; and (4) repeat step 3 until a stopping iteration (chosen via bootstrap or cross-validation) is met. While the algorithm could conceivably run until convergence, a stopping iteration *m*_stop_ is established in order to prevent overfitting and lower prediction accuracy. Tuning *m*_stop_ to some finite value results in an implicit variable selection property, as only one base learner is selected during each iteration. Further, the use of a shrinkage penalty in model fitting provides L1-penalized model coefficients.

Penalization supplies decreased variability of model estimates at the cost of slightly increased bias and helps alleviate problems of collinearity (Kuhn and Johnson, 2013). More complex models with a large number of predictors *P* relative to the number of participants in the sample *N* may have unstable and inflated parameter estimates due to increasing inter-correlations among predictors (collinearity). The mboost algorithm optimizes prediction by removing predictors via variable selection and by using penalization to counter inflated parameter estimates that result from collinearity. The generalized linear/additive model building process also results in readily interpretable models, as opposed to many other ML algorithms that may generate interpretation-resistant or “black box” predictions.

#### Model Reduction

The final optimized model chosen via component-wise gradient boosting features regularized parameter estimates and inherent variable selection. This model may then be simplified to maximize parsimony at the expense of pure predictive power and increased bias in estimation in a process called model reduction. To find the most parsimonious model, we engage in backward elimination from the optimized model fit in mboost. Backward elimination is an exploratory stepwise procedure that begins with all of the variables in the optimized model fit by mboost and tests the fit of the model (measured by Akaike information criteria, or AIC) by the deletion of each variable. The variable (if any) that most improves the model by being deleted is then removed. This process is repeated until further deletion does not improve the model. A simplified model that retains around 95% of the fit (e.g., via *R*^2^) of the full model may be considered a successful approximation (Ambler et al., 2002; Harrell, 2015). Reduction may also result in a model with a more attractive parameter-to-sample size ratio. For the present analysis, backward elimination is performed using the StepAIC() function in the MASS package in R (Venables and Ripley, 2002; R Core Team, 2017).

## Results

Table 1 provides summary statistics for all demographic, psychometric and FKBP5 predictors included in the model. The sample was largely male (77%) and African American (77%). FKBP5 allele distributions did not deviate from Hardy-Weinberg equilibrium. The mean BPAQ score was 64.04 (SD = 19.78, range = 32–111). This is comparable to previous studies in our lab examining individuals with a history of SUD and ASPD (Gowin et al., 2010, 2013; Alcorn et al., 2013). Across those studies, the mean BPAQ value = 67.44 (SD = 15.95, range = 40–124).

### Component-Wise Gradient Boosting

The mboost() function was used to derive an optimal model fitting BPAQ total score to a set of 20 candidate base-learners. Tuning the optimal number of boosting iterations by 10-fold cross-validation resulted in *m*_stop_ = 38. The resultant model retained 8 of the 20 predictors and yielded an *R*^2^ = 0.66. Standardized penalized coefficients for these predictors are included in Table 2. These coefficients included smoking status, FKBP5_13 allelic variants C/T and T/T, and several subscales from the CTQ (trauma) and SRP3 (psychopathy) measures. For this eight-factor model the three strongest predictors were the three retained subscales from the SRP3 psychopathy measure. These measures were related to increases in BPAQ total score of 7.24, 3.27 and 2.25 points for one standard deviation increases in callous affect, ELS and criminal tendencies, respectively.

**Table 2:** Parameter estimates of the optimized model derived by the mboost algorithm, based on the original 20 predictor variables with Buss-Perry Aggression Questionnaire (BPAQ) total score as the outcome variable, ranked by absolute value.

Variable	Coefficient
SRP3_CA	7.238
SRP3_ELS	3.273
SRP3_CT	2.251
CTQ_PN	−2.132
SRP3_IM	1.633
CTQ_PA	1.428
FKBP5_13-2 (T/T)	−0.994
Smoker-1 (YES)	0.722
FKBP5_13-1 (C/T)	−0.251

*Predictors were z-scored before estimation; BPAQ total score was measured in raw units. R^2^ = 0.651. Note: FKBP5_13 = rs1360780; CTQ, Childhood Trauma Questionnaire (PA, physical aggression; PN, physical neglect); SRP3, Self-Report of Psychopathy (IM, interpersonal manipulation; CA, callous affect; ELS, erratic lifestyle; CT, criminal tendencies)*.

### Model Reduction With Elimination

Results of the model reduction using the backwards elimination technique from the full penalized eight-factor model are shown in Table 3. For model comparison purposes, the variables selected by the mboost algorithm were refit in an unpenalized model before backward elimination. Backwards elimination shifted *R*^2^ from 71.8 to 71.4, thus approximating 99.4% of the *R*^2^ (the coefficients from the backward elimination process are unpenalized and yield a different basis for *R*^2^ from the boosted model). The model was highlighted by the following relationships: active smoking was associated with higher trait aggression; having the FKBP5_13 T/T allele was associated with lower trait aggression relative to having the FKBP5_13 C/C allele (reference contrast); CTQ history of childhood physical abuse was associated with higher trait aggression while history of physical neglect was associated with lower aggression; and SRP3 callous affect was associated with higher trait aggression. While model parameters from stepwise selection are inherently biased (coefficients may be inflated), bootstrap standard errors and 95% confidence intervals are provided to ensure maximum possible robustness of statistical inferences. Table 3 describes parameter estimates for the reduced model. The strongest effects found in the reduced model demonstrated that a one standard deviation increase in callous affect was related to a 10.7 point increase in BPAQ total score and that presence of the T/T allele (as compared to the C/C allele) was related to a 10.7 point decrease in BPAQ total score.

**Table 3:** Coefficients, standard errors, *t*-values, *p*-values and bootstrapped SE and 95% confidence intervals from the final simplified six-factor model (adjusted *R*^2^ = 0.66), derived via backwards elimination from the full penalized eight-factor model.

Variable	Estimate	SE	*t* value	*p*-value	Bootstrap SE	Bootstrap 95% CI	
(Intercept)	64.693	2.968	21.799	0.000	3.061	59.610	72.170
SMOKER (YES)	8.386	3.507	2.391	0.021	3.701	1.409	16.030
FKBP5_13-1 (C/T)	−6.006	3.884	−1.546	0.130	4.835	−15.461	3.543
FKBP5_13-2 (T/T)	−10.733	4.910	−2.186	0.035	3.977	−18.620	−3.070
CTQ_PA	6.074	1.897	3.203	0.003	1.694	1.945	8.951
CTQ_PN	−5.395	1.863	−2.896	0.006	1.725	−9.529	−2.195
SRP3_IM	3.316	2.448	1.355	0.183	2.428	−1.200	8.320
SRP3_CA	10.689	2.602	4.107	0.000	3.088	4.910	17.290

*Note: FKBP5_13 = rs1360780 (PA, physical aggression; PN, physical neglect); CTQ, Childhood Trauma Questionnaire; SRP3_CA, Self-Report of Psychopathy Callous Affect*.

## Discussion

The present report used the mboost technique with subsequent backward elimination to determine a parsimonious set of predictors of trait aggression, highlighted by associations with callous affect, childhood trauma and FKBP5_13 alleles. While our analytic approach was not hypothesis-driven, these predictors correspond with the broader extant literature on human aggression. Both childhood trauma and callous unemotional traits are robustly associated with aggression and related conduct problems during adolescence and adulthood (Hare and Neumann, 2009; Frick and Ray, 2015; Milaniak and Widom, 2015; Gillikin et al., 2016). Moreover, there is growing empirical support that the FKBP5 gene plays a key role in the modulation of the stress response and the regulation of emotion, including risk for aggressive behavior (Klengel et al., 2013; Bryushkova et al., 2016), and the present study is the first to demonstrate this relationship in adults, and the first to demonstrate an association between aggression and the T allele of rs1360780. While beyond the scope of the present data, it is possible that the predictive utility of FKBP5 and CTQ abuse variables result from the presence of a gene × environment phenotype (Tuvblad and Baker, 2011).

The mboost technique is a modern hybrid approach that sits in between traditional generalized linear models and ML approaches that model interactions of higher-order complexity (Hothorn et al., 2016). Supervised ML techniques, including ensemble boosting and bagging approaches like mboost (Bühlmann and Hothorn, 2007), offer utility in identifying relationships among complex, multifactorial phenomena that define many human behaviors, such as violence and aggression. Such analytic approaches provide advantages to modern translational research that seeks to integrate across diverse sources of high-dimensional data, for example genetics, neuroimaging and psychometrics. In the present context, these techniques provide automated optimization of a predictive regression model for an outcome of interest, such as aggression. As opposed to traditional statistical analyses, these algorithms can maximize the utility of available data without “data dredging, ” whereby many relationships between variables are examined in an exhaustive yet unsystematic fashion, and only the significant relationships are reported. Such research products represent part of the current controversy surrounding poor replication of findings in the behavioral sciences. Here we fully acknowledge the limitations of the modest amount of available data, using ML to optimize the statistical modeling of that data, and providing incremental knowledge gained. Accordingly, the present findings should reinforce previous evidence that childhood abuse and psychopathic traits increase trait aggression, and should also provide preliminary evidence of relationships between the FKBP5 polymorphism and trait aggression in adults. In particular, the strongest predictors (callous affect, FKBP5_13 T/T allele) were related to approximately 10 point differences in BPAQ total score per standard deviation, as compared to the reference category.

It should be noted that neither the boosting model nor the backwards elimination model should be considered correct. The two complementary models provide different levels of detail regarding the relationships between the predictors and the outcome. To the extent that future samples are similar in nature to the present sample, the penalized boosting model may be a better reference model. Increasingly dissimilar samples may be better represented by the more parsimonious reduced model. Given the high degree of approximated fit obtained here, the reduced model may be sufficient in most contexts; however, this should not be taken to mean that it is superior—only different in applicability.

The limitations of the present project constrain the generality of the results, but they are encouraging in supporting a growing literature linking *FKBP5* expression and exposure to stressors (e.g., childhood trauma) to emotional dysregulation. Dysregulation may be expressed in a variety of behavioral manifestations, including psychopathy (callous affect), deficient inhibitory control, and aggressive behavior. In the present case, we show that T carriers of the *FKBP5* rs1360780 are tied to trait aggression and hostility (BPAQ); the predictive model accounted for approximately 66% (boosting) and 71% (backwards elimination) of the variance. Previous results using similar data science analytic methods obtained prediction outcomes of AUC = 0.76, 0.74 and 0.77 for cardiac events (Wu et al., 2010), methamphetamine relapse (Gowin et al., 2015), and suicide attempts (Passos et al., 2016), respectively. Putting the accuracy of any such model into proper context requires an understanding of not only the accuracy of prior models that addressed phenomenon of similar complexity (i.e., human aggression), but also of the limits of best performance that can reasonably be expected. Such limits may be constrained by insufficient data (e.g., the small sample size available in the present analysis), model sophistication, and in the phenomenon of interest (Hastie et al., 2009; Hofman et al., 2017). This study did not stratify genetic effects by ancestry, which could lead to occult stratification. However, as the sample was predominantly of African ancestry, stratification seems unlikely, although it remains unclear if the effects of FKBP5 on aggression extend to European or Asian ancestry samples. How well these results generalize to broader populations or clinically diagnosed groups is important, and will need to be ascertained in replication studies involving other populations selected based either on specific clinical criteria or obtained from larger, more heterogeneous samples. Accordingly, the value of the present data will be determined by the ability of future projects to systematically replicate the results with extended and enriched samples.

In the present report, we provide a modest example of the application of modern analytic data science techniques (gradient boosting) to data obtained within the context of an experiment that featured a range of variables selected based on the extant literature. Typically, studies of the present kind do not provide for statistical techniques that validly allow simultaneous examination of all factors. However, via this hybrid approach, we show that approximately two-thirds of the variation in trait aggression (BPAQ) was predicted by an initial combination of eight, and subsequently six key variables. Notably, the final model included psychometric personality variables (callous affect), developmental history (childhood trauma) and genetic variants (FKBP5). While cogent accounts of complex, multifactorial interactions require larger, more detailed, and longitudinal datasets, the results underscore the emerging importance of understanding gene × environment interactions in emotional dysregulation and aggression (Tuvblad and Baker, 2011; Weeland et al., 2015; Holz et al., 2016). The current approach and dataset were underpowered to examine such interactions, but such endeavors are currently planned for larger datasets culled from electronic medical records data. Notably, several of the variables under consideration in this project were previously examined in isolation. These individual variables were identified as predictors in independent studies. One novel feature of this project was the examination these factors in the same individuals. Accordingly, the FKBP5, SRP and CTQ data collectively add value by providing systematic (vs. direct) replication of prior findings. Recent work has highlighted the importance of replication in science (i.e., “reproducibility”; Aarts et al., 2015; Elliott and Resnik, 2015). Here, we provide preliminary data suggesting these variables are collective predictors of trait aggression.

Access to electronic healthcare system, collaborative multisite and national longitudinal databases has become more common. Accordingly, big data science approaches continue to refine the methods needed to model the complexity in these datasets, and—*critically*—to interpret the outcomes (Dipnall et al., 2016; Krystal et al., 2017; Wiens and Shenoy, 2018). These rapidly developing tools stand to provide deeper understanding of the relationships among neural, genetic, psychological, and contextual variables in human aggression, moving toward improved prediction and prevention efforts.

## Author Contributions

RS performed the primary statistical analyses (component-wise gradient boosting, model approximation) and co-wrote the data analytic strategy and results. JLG helped conceive and develop the original experiments and helped author with the introduction and methods. CEG guided the statistical approach and co-authored the data analytic strategy. CW-B processed and analyzed all the saliva samples to derive the genetic data for the FKBP5 SNPs. SDL helped conceive and develop the original experimental design, and served as senior author on the project, providing oversight over the project and each section of the manuscript.

## Conflict of Interest Statement

The authors declare that the research was conducted in the absence of any commercial or financial relationships that could be construed as a potential conflict of interest. The handling Editor declared a past co-authorship with one of the authors SDL.
